# The impact of COVID-19 non-pharmaceutical interventions on the lived experiences of people living in Thailand, Malaysia, Italy and the United Kingdom: A cross-country qualitative study

**DOI:** 10.1371/journal.pone.0262421

**Published:** 2022-01-21

**Authors:** Mira L. Schneiders, Bhensri Naemiratch, Phaik Kin Cheah, Giulia Cuman, Tassawan Poomchaichote, Supanat Ruangkajorn, Silvia Stoppa, Anne Osterrieder, Phee-Kheng Cheah, Darlene Ongkili, Wirichada Pan-ngum, Constance R. S. Mackworth-Young, Phaik Yeong Cheah

**Affiliations:** 1 Mahidol Oxford Tropical Medicine Research Unit, Faculty of Tropical Medicine, Mahidol University, Bangkok, Thailand; 2 Nuffield Department of Medicine, Centre for Tropical Medicine & Global Health, University of Oxford, Oxford, United Kingdom; 3 Nuffield Department of Population Health, Ethox Centre, Big Data Institute, University of Oxford, Oxford, United Kingdom; 4 Faculty of Arts & Social Science, Universiti Tunku Abdul Rahman, Kampar, Malaysia; 5 Paediatric Ethics Committee; Research Ethics Committee, University Hospital of Padua, Padua, Italy; 6 The SoNAR-Global Network, Mahidol University, Bangkok, Thailand; 7 Luoghi di Prevenzione, Reggio Emilia, Italy; 8 Emergency and Trauma Department, Sabah Women and Children’s Hospital, Ministry of Health Malaysia, Kota Kinabalu, Malaysia; 9 Emergency and Trauma Department, Queen Elizabeth Hospital, Ministry of Health Malaysia, Kota Kinabalu, Malaysia; 10 Department of Tropical Hygiene, Faculty of Tropical Medicine, Mahidol University, Bangkok, Thailand; 11 Department of Global Health and Development, London School of Hygiene and Tropical Medicine, London, United Kingdom; University of Toronto, CANADA

## Abstract

This qualitative study explores the impact of non-pharmaceutical interventions (NPIs), including social distancing, travel restrictions and quarantine, on lived experiences during the first wave of the COVID-19 pandemic in Thailand (TH), Malaysia (MY), Italy (IT) and the United Kingdom (UK). A total of 86 interviews (TH: n = 28; MY: n = 18; IT: n = 20; UK: n = 20) were conducted with members of the public, including healthcare workers (n = 13). Participants across countries held strong views on government imposed NPIs, with many feeling measures lacked clarity. Most participants reported primarily negative impacts of NPIs on their lives, including through separation, isolation and grief over missed milestones; work-related challenges and income loss; and poor mental health and wellbeing. Nonetheless, many also experienced inadvertent positive consequences, including more time at home to focus on what they most valued in life; a greater sense of connectedness; and benefits to working life. Commonly employed coping strategies focused on financial coping (e.g. reducing spending); psycho-emotional coping (e.g. engaging in spiritual practices); social coping and connectedness (e.g., maintaining relationships remotely); reducing and mitigating risks (e.g., changing food shopping routines); and limiting exposure to the news (e.g., checking news only occasionally). Importantly, the extent to which participants’ lived experiences were positive or negative, and their ability to cope was underpinned by individual, social and economic factors, with the analysis indicating some salient differences across countries and participants. In order to mitigate negative and unequal impacts of NPIs, COVID-19 policies will benefit from paying closer attention to the social, cultural and psychological—not just biological—vulnerabilities to, and consequences of public health measures.

## Introduction

*“We are like islands in the sea*, *separate on the surface but connected in the deep*.*”* William James

In 2020, countries around the world implemented a range of containment measures using non-pharmaceutical interventions (NPIs) to prevent the transmission of SARS-CoV-2 (COVID-19), which to date has resulted in 113,467,303 confirmed cases, including 2,520,550 deaths worldwide [1]. These included social distancing measures, such as contact tracing, quarantine, closure of schools, work places and non-essential shops; travel-related measures such as travel restrictions, screening and border closure; and personal protective measures such as face masks, hand hygiene and respiratory etiquette [2]. Evidence supports the effectiveness of these measures in reducing COVID-19 transmission, mortality and healthcare demand [3–6].

Despite this evidence, ethical considerations need to account for the economic, social and psychological impacts of NPIs on people’s lives [7]. For this, it is vital to understand how NPIs are experienced by the public. A systematic review and synthesis of qualitative studies on public perceptions on NPIs in the context of respiratory infections found that perceptions were impacted by assessments about their necessity, efficacy, acceptability, and feasibility [8]. Furthermore, the potential to attract stigma, perceptions about the emotional, personal and societal economic costs, and cultural unacceptability were identified as disadvantages of social distancing, isolation and quarantine [8].

The importance of focusing on mental health amidst this pandemic has been underscored by the emergence of evidence highlighting the negative psychological impacts of NPIs [9]. A recent review on the psychological impact of quarantine identified post-traumatic stress symptoms, confusion, and anger as negative effects, and found that longer quarantine duration, infection fears, frustration, boredom, inadequate information and supplies, financial loss, and stigma were important stressors [10]. While these findings do not suggest that NPIs, like quarantine and isolation should not be employed, the adverse psychological impacts they may cause need to be weighed against likely benefits [10].

Emerging evidence also suggests that the use of NPIs in the context of COVID-19 has had unequal effects on society, including across and within countries. For example, a survey comparing the impact of NPIs in Thailand, Malaysia, Italy, Slovenia and the UK found that those living in lower income countries reported more severe economic impact during the first wave of COVID-19 restrictions [11]. Another survey conducted among 17 countries in Latin America and the Caribbean found that the poorest households reported the highest levels of job loss and household food insecurity and that these impacts were also substantially higher among countries with mandatory NPIs, such as quarantines, curfews and mobility restrictions, compared to countries not implementing such restrictions [12]. Additionally, vulnerable populations in particular, including migrants, people working in the informal economy, homeless people, older people and people living with disabilities, may be disproportionately affected by the adverse impacts of NPIs [7, 13]. Vulnerable individuals may be less able to comply with NPIs, due to underlying inequalities in the distribution of resources, wealth and power [13–15]. For example, they may be less able to practice hand hygiene (if lacking access to basic sanitation), wear masks (due to access and affordability issues) or adopt recommended social distancing (when living in crowded conditions, relying on public transport, working in high public contact jobs or being unable to access sick leave) [13–15]. This, in turn is likely to exacerbate existing health, economic and social inequalities.

Understanding the social, economic and mental health impacts of prolonged exposure to social distancing and isolation during the COVID-19 pandemic has been identified as a priority for multidisciplinary COVID-19 research [16]. The importance of doing this research “together with people with lived experience” has also been highlighted [17]. Qualitative research in particular can help to gain a rich and comprehensive understanding of lived experience [18]. Knowledge generated using social science methods is thus crucial for generating insights that can inform the development of more effective and equitable public health interventions in light of infectious disease threats [19].

To date, little qualitative research has been conducted to understand public perceptions and lived experiences of NPIs. A focus group study conducted in the UK found that feelings of anxiety, depression and loss were common in response to COVID-19 social distancing and isolation [20]. However, the study did not include healthcare workers (HCW) or people at higher-risk of severe illness from COVID-19, such as those with underlying chronic conditions or over the age of 60 years [21], and so little is known about how these groups are impacted by NPIs. While no qualitative studies have directly studied the impact of NPIs on HCW, existing quantitative evidence suggests that HCW are at greater risk of experiencing psychological distress during the COVID-19 outbreak, including symptoms of depression, anxiety and PTSD [22–25]. Furthermore, to our knowledge, no qualitative study has been conducted comparing lived experiences of NPIs across different countries and world regions. Cross-national studies are useful because they can facilitate understanding of common issues [26] and generate deeper insights into the phenomena being studied [27]. This study therefore aims to help address these gaps. Using in-depth interviews, we seek to explore and compare the lived experiences, coping strategies and views of government imposed COVID-19 NPIs among the public and HCW across four countries, namely Thailand, Malaysia, Italy and the United Kingdom (UK).

## Materials and methods

### Study design

This study used a phenomenological approach, focused on “the study of an individual’s lived experiences within the world” [28]. In-depth, semi-structured interviews were conducted with 86 individuals residing in four countries: Thailand (TH), Malaysia (MY), Italy (IT) and the UK (UK) between May 2 and August 4 2020 (TH: 8.05–21.07.2020; MY: 2.05–4.07.2020; IT: 13.05–04.08.2020; UK: 14.05–23.07.2020). Countries were selected based on the implementation of COVID-19 public health measures (NPIs) during the study period and the existence of prior research collaborations, which enabled and facilitated timely implementation of the study. Here we describe results from a qualitative study, which was part of a mixed-methods study (SEB-COV) that also included an online survey [29]. The quantitative results are reported elsewhere [11].

### COVID-19 government response in the four countries

**Fig 1:**
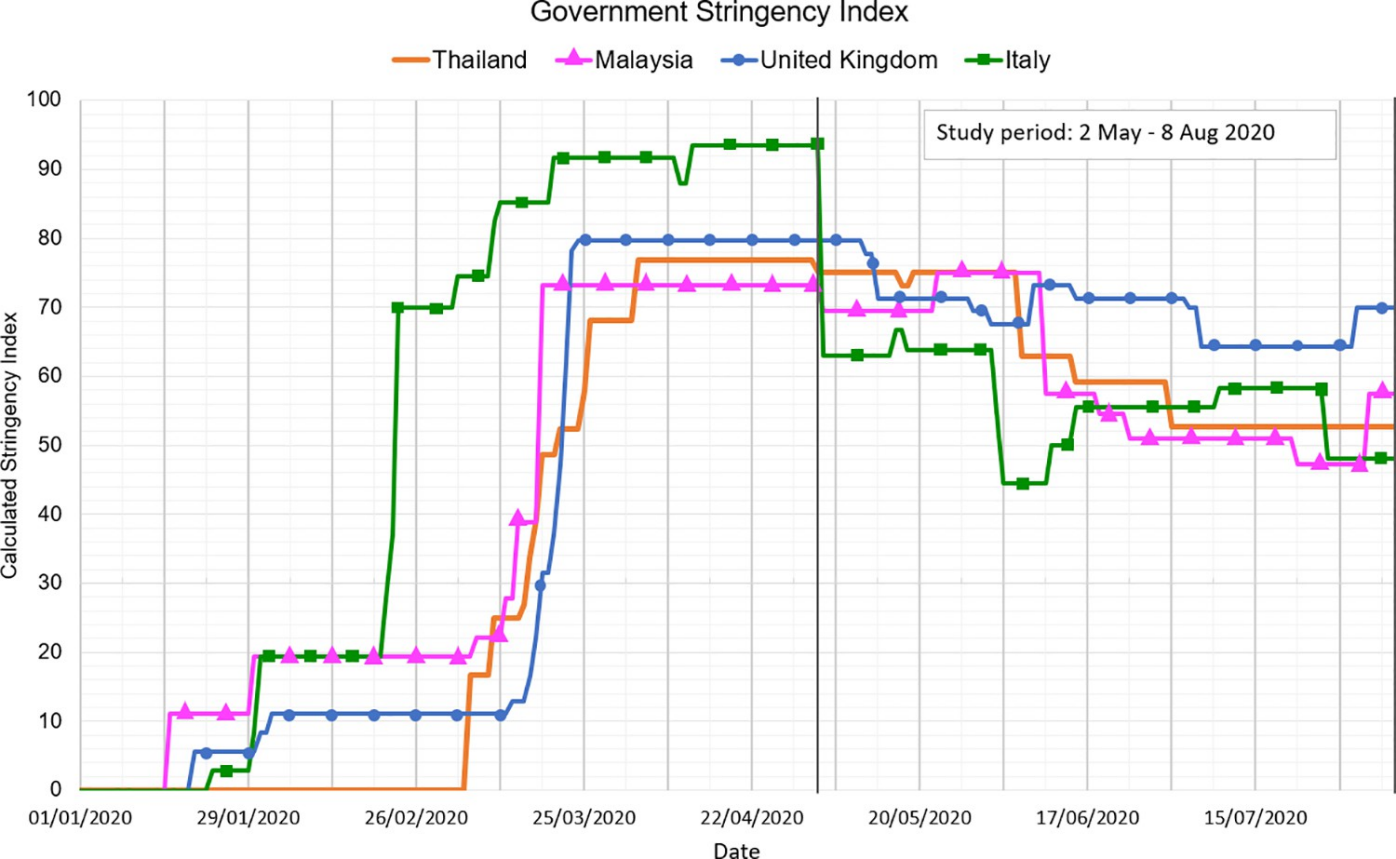
Government stringency index. SI during the first wave of COVID-19 NPIs for Thailand, Malaysia, Italy and the UK.

Over the study period, Thailand, Malaysia, Italy and the UK were under varying degrees of lockdown to prevent the transmission of COVID-19, which included closures of workplaces and schools, restrictions on movement and travel, and social distancing guidelines. The Oxford COVID-19 Government Response Tracker (OxCGRT) [30] tracks government responses to COVID-19 from more than 180 countries on standardized indicators. Data are aggregated to produce a daily ‘Stringency Index’ (SI), on a scale from 0–100, with 100 indicating the strictest public health response. Fig 1 visualises the SI of the four governments over the study period. For example, Italy experienced the strictest restrictions at the beginning of May (SI = 93), followed by easing of restrictions in early June (SI = 44) and subsequent tightening. In Thailand, the SI was high at the start of the study (SI = 77) and decreased at the end of June (SI = 53). The pattern was similar in Malaysia. Restrictions in the UK remained high throughout (SI = 69–76).

Furthermore, the socio-political context within which each country has experienced the pandemic and the measures introduced to curb COVID-19 transmission provide important background to this study.

Italy and the UK are both high-income countries in Europe [31]. Serving a population of 60.4 million [32], the Italian National Healthcare Service (Servizio Sanitario Nazionale) is comprised of regionally based, decentralised health services. Despite Universal Health Coverage, the quality of healthcare services varies by region, with interregional differences exacerbated during the COVID-19 pandemic [33]. Italy was the first country outside of China to report cases of COVID-19, recording among the highest numbers of COVID-19 cases in the world in early 2020 and overtaking cases in China on 19 March 2020 [34, 35]. Despite an epidemiological north-south divide, with northern regions being worst affected and hospitals running over capacity, the Italian government introduced a nationwide homogenous lockdown on 9 March 2020 lasting until 4 May 2020 [35], comprising measures to close down non-essential businesses, ban private gatherings, and restrict movement within cities and between regions, with people only allowed to leave their home for essential reasons (e.g. work, grocery shopping, health-related problems) [36]. A detailed analysis of the Italian COVID-19 response has been provided elsewhere [35, 37].

In the UK, the National Health System (NHS) provides Universal Health Coverage free at the point of care to the population of 67.8 million [32], through primary- (community care, GPs, pharmacies), secondary- (hospital-based care via GP referral) and tertiary-level care (specialist hospitals) [38]. On 23 March 2020, a UK-wide national lockdown was announced [39, 40], with closure of schools and non-essential businesses and ‘stay-at-home orders’ restricting residents from leaving their home without a ‘reasonable excuse’, such as shopping for necessities, conducting essential work, and exercise. UK Government guidance on social distancing, published on March 30, provided advice on avoiding non-essential contacts and maintaining two-meters distance to other people [41] and further ‘shielding’ guidance were introduced to protect individuals identified as clinically extremely vulnerable [42]. On May 13, 2020, the four UK nations implemented various phased approaches to lift lockdown restrictions, including the amendment of social distancing guidelines to one-meter in England and Northern Ireland [43]. By July 14, 2020, the UK was the worst affected country in Europe with over 290,000 COVID-19 cases and over 44,000 deaths [44], primarily in England [45]. To mitigate the impact of NPIs, the government introduced various economic support schemes for individuals and businesses, including the furlough scheme and payments for low income families [46]. A detailed summary of the UK national COVID-19 response is provided elsewhere [47].

Thailand and Malaysia are both upper-middle-income countries in South-East Asia [31]. Since 2002, Thailand has had Universal Health Coverage [48] serving its population of 69.7 million [32]. While the majority of healthcare is delivered via the public sector, one quarter of Thai hospitals are private [48]. In response to COVID-19, the governmental Centre for COVID-19 situation Administration (CCSA) was established to take responsibility for the national pandemic response, management, and treatment of COVID-19 cases [49]. On 21 March 2020, the Governors of Bangkok, and other key affected provinces in Thailand imposed an emergency decree to ensure social distancing, including closing of retail businesses. On 3 April 2020, border closures and a nationwide curfew was announced, with all residents instructed to remain inside their homes between the hours of 10pm-4am and to wear masks outside their homes. On 3 May 2020, a phased approach to relax these measures was introduced, with schools and selected businesses reopening on 1 July 2020, while high-risk businesses including bars, massage parlours and children’s play areas remaining closed. To support Thailand’s 3 million informal workers, the government introduced a financial support scheme in April 2020, providing 5000 Baht cash handouts for up to three months, emergency loans and electricity bill subsidies [50]. A detailed analysis of Thailand’s COVID-19 response is provided here [51].

Malaysia’s population of 32.8 million [32] comprises multi-ethnic and multi-religious groups, with different beliefs, traditions and health related practices. The healthcare system is regulated by the federal government, consisting of both private healthcare providers and more accessible and affordable public hospitals and clinics. On 25 January 2020, the first COVID-19 case in Malaysia was reported [52]. Following a mass religious gathering in February 2020, Malaysia reported the highest number of COVID-19 cases in South-East Asia [53], with the Malaysian Ministry of Health (MOH) declaring a Movement Control Order between 16 March 2020 and 28 April 2020, prohibiting mass movements and gathering, closing non-essential businesses, places of worship, schools and borders, restricting cross-state travel and allowing only one person per household to leave the home to buy essential goods [52, 54]. With a total of 5,251 COVID-19 cases were reported on 17 April 2020 [54], the country was split into different zones depending on geographic variation in case numbers, with enhanced measures and COVID-19 screening in place in highly effected areas [52]. To reduce the economic impact of NPIs, the government introduced the PRIHATIN Economic Stimulus Package, including one-off payments to support small businesses and low income households [55]. More details on the Malaysian COVID-19 response are provided elsewhere [52, 55].

### Participant selection

All participants talking part in the SEB-COV online survey [29] were invited to take part in the qualitative arm of the study. Additionally, in each country, participants were recruited through existing research and organisational networks, social media channels (Facebook, Twitter), and targeted social media advertising (Facebook adverts). To encourage greater diversity among the sample, key community organisations and institutions working with vulnerable, hard-to-reach, or high-risk populations (e.g., entertainment workers, family caregivers, frontline workers, older people) were contacted via email and asked to disseminate information about the study through their networks.

Individuals were provided with information about the study and asked to register their interest using a short socio-demographic recruitment survey online, administered using the ‘JISC online surveys’ platform [56]. Across countries, a total of 276 participants registered their interest via the recruitment survey (TH: 34; MY: 52; IT: 34; UK: 156). Among these, participants were selected by each country team using purposive sampling to gain a maximum variation sample, based on six characteristics (i.e., gender, age, number of household members, self-perceived level of risk, level of education and occupation). Selected individuals were invited to take part in an interview via email or telephone (total 127, TH: 29; MY: 24; IT: 34; UK: 40). Of those contacted, 91 individuals agreed to participate, with five dropping out prior to the interview. A total of 86 interviews were therefore conducted across the four countries.

Participants in Thailand received ฿200 THB (USD 7), and in the UK were provided a £10 electronic supermarket voucher to compensate for their time. No compensation was provided in Italy and Malaysia, in accordance with local research practices.

### Participant characteristics

A total of 86 participants took part in an in-depth interview (n = 20 UK, n = 28 Thailand, n = 18 Malaysia, n = 20 Italy), with 51% of all participants being female, 43% male and 6% identifying as other genders (Table 1). Participant age ranged from 18–84 years (IT:20–65; MY 19–84; TH 18–74; UK:24–80 years), with a total of 12.8% (n = 11) aged 65 years or older. Participants varied in their levels of education (5% primary, 31% secondary, 64% tertiary), household size (range: 1–12 people) and occupations (15% HCW, 85% other). Across all countries, 41% of participants perceived themselves at low, 37% at medium and 22% at high risk of COVID-19.

**Table 1:** Participant characteristics.

Characteristic	United Kingdom	Thailand	Malaysia	Italy	Total
	**n (%)**	**n (%)**	**n (%)**	**n (%)**	**n (%)**
**Gender**	n = 20	n = 28	n = 18	n = 20	n = 86
Female	12 (60.0)	12 (42.9)	8 (44.4)	12 (60.0)	44 (51.2)
Male	8 (40.0)	11 (39.3)	10 (55.6)	8 (40.0)	37 (43.0)
Other	0 (0.0)	5 (17.9)	0 (0.0)	0 (0.0)	5 (5.8)
**Age range**					
18–24	1 (5.0)	2 (7.1)	1 (5.6)	2 (10.0)	6 (7.0)
25–34	2 (10.0)	5 (17.9)	3 (16.7)	8 (40.0)	18 (20.9)
35–44	1 (5.0)	12 (42.9)	4 (22.2)	2 (10.0)	19 (22.1)
45–54	4 (20.0)	4 (14.3)	5 (27.8)	5 (25.0)	18 (20.9)
55–64	6 (30.0)	3 (10.7)	3 (16.7)	2 (10.0)	14 (16.3)
65–74	5 (25.0)	2 (7.1)	1 (5.6)	1 (5.0)	9 (10.5)
75–84	1 (5.0)	0 (0.0)	1 (5.6)	0 (0.0)	2 (2.3)
**Highest level of education**					
Primary	0 (0.0)	4 (14.3)	0 (0.0)	0 (0.0)	4 (4.7)
Secondary	5 (25.0)	9 (32.1)	6 (33.3)	7 (35.0)	27 (31.4)
Tertiary	15 (75.0)	15 (53.6)	12 (66.7)	13 (65.0)	55 (64.0)
**Number of household members**					
1	6 (30.0)	5 (17.9)	2 (11.1)	4 (20.0)	17 (19.8)
2	7 (35.0)	7 (25.0)	4 (22.2)	3 (15.0)	21 (24.4)
3	4 (20.0)	6 (21.4)	4 (22.2)	9 (45.0)	23 (26.7)
4	2 (10.0)	4 (14.3)	1 (5.6)	2 (10.0)	9 (10.5)
5	0 (0.0)	3 (10.7)	5 (27.8)	2 (10.0)	10 (11.6)
6	0 (0.0)	1 (3.6)	0 (0.0)	0 (0.0)	1 (1.2)
7	1 (5.0)	1 (3.6)	1 (5.6)	0 (0.0)	3 (3.5)
>7	0 (0.0)	1 (3.6)	1 (5.6)	0 (0.0)	2 (2.3)
**Overall self-perceived risk level**					
Low	9 (45.0)	8 (28.6)	4 (22.2)	14 (70.0)	35 (40.7)
Medium	7 (35.0)	13 (46.4)	8 (44.4)	4 (20.0)	32 (37.2)
High	4 (20.0)	7 (25.0)	6 (33.3)	2 (10.0)	19 (22.1)
**Occupation** ^**a**^					
**1 Managers**^**b**^	**2 (10.0)**	**2 (7.1)**	**6 (33.3)**	**0 (0.0)**	**10 (11.6)**
**2 Professionals**^**c**^	**8 (40.0)**	**5 (17.9)**	**3 (16.7)**	**9 (45.0)**	**25 (29.1)**
**3 Technicians and Associate Professionals**^**d**^	**3 (15.0)**	**2 (7.1)**	**3 (16.7)**	**2 (10.0)**	**10 (11.6)**
**4 Clerical Support Workers**^**e**^	**0 (0.0)**	**7 (25.0)**	**0 (0.0)**	**0 (0.0)**	**7 (8.1)**
**5 Service and Sales Workers**^**f**^	**0 (0.0)**	**4 (14.3)**	**0 (0.0)**	**0 (0.0)**	**4 (4.7)**
**7 Handicraft and Printing Workers**^**g**^	**0 (0.0)**	**0 (0.0)**	**0 (0.0)**	**2 (10.0)**	**2 (2.3)**
**8 Plant and Machine Operators and Assemblers**^**h**^	**0 (0.0)**	**3 (10.7)**	**0 (0.0)**	**0 (0.0)**	**3 (3.5)**
**9 Elementary Occupations**^**i**^	**0 (0.0)**	**1 (3.6)**	**0 (0.0)**	**2 (10.0)**	**3 (3.5)**
** Others (not specified in ISCO-08)**	**7 (35.0)**	**4 (14.3)**	**6 (33.3)**	**5 (25.0)**	**22 (25.6)**
University student	0 (0.0)	2 (7.1)	2 (11.1)	2 (10.0)	6 (7.0)
Retired	5 (25.0)	2 (7.1)	3 (16.7)	2 (10.0)	12 (14.0)
Unemployed	2 (10.0)	0 (0.0)	1 (5.6)	1 (5.0)	4 (4.7)
**Occupational category**					
Healthcare worker	4 (20.0)	4 (14.3)	2 (11.1)	3 (15.0)	13 (15.1)
Non-healthcare worker	16 (80.0)	24 (85.7)	16 (88.9)	17 (85.0)	73 (84.9)

^a^ Occupations have been classified according to the International Standard Classification of Occupations 08 (ISCO-08) [99].

^b^ e.g.: Business Services and Administration Managers; Legislators and Senior Officials.

^c^ e.g.: Other Health Professionals and Other Health Professionals; Social and Religious Professionals.

^d^ e.g.: Legal, Social and Religious Associate Professionals; Government Regulatory Associate Professionals.

^e^ e.g.: General Office Clerks.

^f^ e.g.: Travel Attendants, Conductors and Guides; Hairdressers, Beauticians and Related Workers.

^g^ e.g.: Handicraft Workers.

^h^ e.g.: Car, Van and Motorcycle Drivers.

^i^ e.g.: Domestic, Hotel and Office Cleaners and Helpers; Agricultural, Forestry and Fishery Labourers.

### Data collection

In-depth interviews were used to encourage participants to narrate their own lived experiences of government imposed NPIs. The interview topic guide, which was developed collaboratively in a series of online meetings involving all interviewers from across the four countries was used to interview participants (see S1 File). The interview guide was based on the research aims and protocol of the SEB-COV mixed-methods study [29]. Following pilot testing in each country, the guide was subsequently discussed and refined to focus on three broad areas, namely: (1) lived experiences and perceptions of COVID-19 measures (i.e., social isolation, social distancing, travel restrictions, quarantine); (2) wellbeing and mental health; (3) information, misinformation and rumours (for topic guide see [57]). In Thailand and Italy, topic guides were translated into Thai and Italian respectively, and interviews were conducted in these languages. In Malaysia and the UK, interviews were conducted in English.

Written informed consent was obtained electronically and permission was sought to audio record interviews. In Thailand and Malaysia, all interviews were recorded and transcribed. In Italy and UK, interviewers took detailed notes during the interview and wrote up summary scripts directly after, with salient quotes being transcribed verbatim from the recordings. The interview script method has been shown to produce levels of detail comparable to audio-recorded transcription [58, 59], and was chosen for reasons of time and resource efficiency. Because of data protection regulations preventing re-contacting of participants, transcripts and summaries were not returned to participants for validation and feedback. Interviews were scheduled at a time convenient to participants and lasted between 30–90 minutes.

As COVID-19 regulations prevented meeting face-to-face, the vast majority of interviews in all four countries were conducted by telephone or an online videoconferencing platform (Microsoft Teams, using video if possible), based on participants’ preferences. Following easing of local restrictions, one interview in Thailand and four interviews in Italy were conducted face-to-face at participants’ request, while adhering to local social distancing guidelines.

Only the researcher and participant were present during interviews and participants were encouraged to signal to the researcher if they felt that their privacy was compromised at any time (e.g., by the arrival of family members). No participants indicated this.

### Research team and reflexivity

In each country, interviews were conducted by a team of trained qualitative researchers (Thailand: BN, SR, TP; Malaysia: PKC, TS; Italy: GC, SS; UK: MLS, CMY; SR = male, all others = female), who began by introducing themselves and briefed participants about the aims of the study. Interviewers did not know participants before the study onset. All interviewers were residents in their study country, were affiliated with a national research institution, and fluently spoke the interview language.

### Data analysis

Data was collected iteratively, with emerging insights from each interview used to inform subsequent interviews, and continued until reaching ‘thematic saturation’, the point at which no salient new themes were deemed to emerge within each country [60]. Following completion of data collection, thematic analysis was used to code data into themes and sub-themes, based on the Framework Method [61]. The Framework Method is a tool commonly used in multi-disciplinary health research, offering “a systematic and flexible approach to analysing qualitative data”, particularly amongst large teams [61]. It was chosen because of its efficiency and appropriateness for analysing data collected for pre-defined aims and objectives [62]. Based on the research aims and discussions within the cross-country research team, a broad, deductive set of thematic codes, including primary and secondary level codes, was developed.

The coding framework was tested, refined and subsequently applied to code data in each country. Coded data was then charted into the Framework matrix, which comprised three major themes (1. Views on public health measures; 2. Lived experience and impact of NPIs; 3. Coping strategies), and additional subthemes. Regular online meetings between the lead researcher (MLS) and country teams were used to review and discuss interpretations and coding of data, as well as charting of interview data into the Framework matrix. Finally, key findings for each theme–both across participants and across countries–were discussed and consolidated in online coding workshops between the lead researcher and country teams. Using the framework matrix, each country team then summarised the key emerging findings for each theme, which served as the basis for cross country comparison. Regular team meetings supported a diversity of perspectives on data analysis, helping to strengthen reliability and validity of the findings [63].

### Ethical approval

Ethics approval was granted by Oxford Tropical Research Ethics Committee (OxTREC, reference no.520-20), covering all four countries. Additional ethics committee approval from Italy was not required for the study to be conducted there. In Thailand, additional approval was sought from the Faculty of Tropical Medicine Ethics Committee (FTMEC, ref: MUTM 2020-031-01). In Malaysia, additional approval was granted by the Medical Research and Ethics Committee (MREC), the Ministry of Health Malaysia (MOH ref: NMRR-20-595-54437 (IIR)), and the Universiti Tunku Abdul Rahman (UTAR) Scientific and Ethical Review Committee (SERC), ref: (U/SERC/63/2020).

## Results

Below we summarize key findings for each of the three major themes discussed. Supporting quotes which are referenced below (e.g., Q1, Q2, Q3 etc) are presented in Table 2.

**Table 2:** Supporting quotes from in-depth interviews conducted across four countries.

Ref	Country and respondent information	Quote
**1. Views on COVID-19 public health measures**		
***Agreement with NPIs and government response***		
**Q1**	Thailand *(#6*, *male*, *42*, *Food delivery driver)*	“I totally agree with the government measures. If government doesn’t do this, I am sure [Thailand] would have a lot of cases. Though I think our government has implemented these measures a bit late, there were some misunderstandings at the beginning, and it was quite strict at first. But overall, I think it is very good and causes lower death and cases in Thailand.”
**Q2**	Malaysia *(#7*, *female*, *49*, *Lecturer)*	“…I support and agree to this decision [to lockdown] because this is a form of control. […] if the government doesn’t do this, we may end up like Italy or America. […] It doesn’t depend on the technology or modernization, it’s more [about] the decision. If your decision is a good one, then you’re safe.”
**Q3**	Italy, *(#6*, *female*, *32*, *Shop assistant)*	“There was unity, and we are all in the same boat.”
***Criticism of NPIs and government response***		
**Q4**	Malaysia *(#8*, *male*, *32*, *Admin Officer)*	“…[The measures are] not strict enough. [The government] should have made it strict until the end, until it settles down, then only they release. […] they worry about their reputation as well. That’s why they start to loosen the restrictions. I will say it is risky because there are still some cases going around. […] So, everything is not under control yet, because you can see in China, the numbers go very low, but once they loosen the restrictions, somehow it spiked again.”
**Q5**	UK *(#15*, *male*, *55*, *IT manager)*	“I was tracking the virus sort of from January and reading about it. It was pretty obvious to me that it was coming our way. […] And I feel we locked down way too late, so we actually withdrew our children from school early […]. I think we locked down at least two weeks too late and many lives could have been saved if we locked down sooner. I think at the other end we’re releasing too early […]. And I find it very strange that we’ve been so slow to, to not even make masks mandatory really.”
**Q6**	UK *(#6*, *male*, *53*, *Social worker)*	“So, before we have even come off the [current] wave, we’re going to go in the second wave but the government have got used to playing with the numbers… every single step along the way has just been a propaganda move and a deception move rather than enabling anybody to be safe. They have caused tens and tens of thousands of deaths and we will easily go over the 100,000 with this second wave, I’m certain of it.**” **
**Q7**	UK *(#15*, *male*, *55*, *IT manager)*	“I am not in an official shielding group so again that’s where I don’t necessarily agree… I’ve had asthma since I was a child. . . I do have reduced lung function. And I’m over 50… My parents are in their mid to late 80s are also not in a shielding group according to the government, which is quite insane, especially as my mum has early-stage dementia… And there has been a linkage suggested between people with Alzheimer’s and susceptibility to COVID. So very poor shielding decisions in my opinion. And absolutely insane that they’re relaxing them at the moment with an arbitrary date of August 1^st^… The virus doesn’t have a calendar. It doesn’t discriminate in those ways.”
**Q8**	Italy *(#5*, *female*, *26*, *Nursing home staff)*	“Some [measures] I think are preposterous because people have been pushed like animals into cages and now that the green light begins [i.e., restrictions are being eased] some people continue to keep the rules, others think that it is all over.”
**Q9**	Thailand *(#22*, *transgender*, *35*, *Interpreter)*	“The Government ฿5,000 [125 USD, per month for 3 months] scheme is not enough at all. How could someone who completely lost their job live on ฿5,000 a month. I have a sick mother that I need to look after. Luckily, I have some financial support from my partner, otherwise, it is impossible to live on ฿5,000 a month.”
**Q10**	Malaysia *(#8*, *male*, *32*, *Admin officer)*	“I did get the aid from government and… it’s not too much. The amount is just few hundred (Ringgit). Not so helpful for me.”
**Q11**	Thailand *(#10*, *female*, *73*, *Retired)*	“The banking system for receiving support money from the government only permitted the use of a government bank. Therefore, many people gathered at the bank, and that increased the risk of spreading and contracting COVID-19.”
**Q12**	UK *(#1*, *female*, *56*, *Photographer)*	“At the moment it all feels very unclear… [the government] is like a parent who is not there. . . I mean [the government’s] job really should be to keep us safe, with the priorities [being] people. But they’re not. And we’re all scrambling around trying to keep us safe and our family safe. And there are so many mixed messages[. . .]. I think what would be most helpful is a feeling of the government putting people first, having really clear guidelines, really clear rules… that are not wishy washy, that this is what we have to do.”
**Q13**	UK *(#3*, *female*, *56*, *Doctor)*	"I don’t trust information from politicians, because I believe they are being driven purely by economic reasons rather than public health reasons."
**2. Lived experiences and impact of public health measures**		
***Positive experiences***		
***More time at home to focus on family*, *oneself and the essential***		
**Q14**	Italy *(#11*, *female*, *30*, *Teacher)*	“I am able to stay at home with my children, enjoy them and see them grow up in these three months.”
**Q15**	Thailand *(#2*, *male*, *31*, *Hotel staff)*	“Sometimes, distancing oneself from other people like this helps to save a lot of money from travel cost to and from work, eating out, or recreational costs. I also have more time for myself and my family. At least, [COVID-19] has some positive aspects.”
***Sense of greater connectedness and shared humanity***		
**Q16**	Italy *(#14*, *female*, *52*, *Social worker)*	“The mood was like ‘you can’t do it alone; you need the help of others. It’s a pain we all feel, so we all have to help each other, behaviours are always interconnected.”
**Q17**	Malaysia *(#3*, *male*, *52*, *Customs officer)*	“My parents have good neighbours who also helped them to buy groceries, and sometimes cooked for them… The temple did some charity work to distribute foodstuff to people who lost their jobs.”
**Q18**	Thailand *(#18*, *female*, *45*, *Dentist)*	‴Tu pan suk’ [sharing food pantries] fit Thai people as it is a way of ‘tum bun’ [making merit/ living virtuously].”
**Q19**	Italy *(#14*, *female*, *52*, *Social worker)*	“Nature has regained its space, there has been a rediscovery of the beauty of nature. We stopped to look at things with a different perspective.”
***Benefits to working life***		
**Q20**	UK *(#18*, *female*, *40*, *Finance officer)*	“Working from home and being able to switch down and switch off certain things has been helpful because I wouldn’t have been able to have that control if I had been at work. And the fact that I am not commuting… is saving me so much time and I honestly prefer it. […] I can achieve everything that I do in the office, but here. […] And I actually dread more when we go back. When restrictions are lifted, I am more concerned about that, and my mental wellbeing and my coping strategies for then, than I am at the moment, to be perfectly honest.”
**Q21**	Thailand *(#1*, *male*, *55*, *Tuk-tuk driver)*	“There is no traffic, I can get home fast and there are barely any cars. In my village there used to be a lot of cars, but now it’s very calm, and at 10pm there are no more cars.”
**Q22**	Thailand *(#24*, *non-binary gender*, *26*, *tour guide)*	“[During the lockdown] I started a small online business selling chili paste made by my mum and my sister… It is going very well. I am now doing my own brand.”
**Q23**	UK *(#5*, *male*, *33*, *Doctor)*	“…Previously I was working a lot harder. I’ve spent most of my working life really burnt out, working for the NHS. I wish I could tell [people clapping on the streets] that I was being a hero at the time. None of the endless night shifts that I previously did were recognised, were clapped.”
***Negative experiences***		
***Separation from family*, *friends and communities*, *and grief over missed milestones***		
**Q24**	UK *(#8*, *female*, *63*, *Retired)*	“…For me [the hardest thing is], not being able to have a hug. I have not had any physical touch whatsoever for 12 weeks […]. People who have got a partner at home or families, I don’t think they understand, you know, how hard that is. […] I’ve become a little bit desensitised to it and it’s just become so normal. I can’t imagine what it’s like to be hugged to be honest. So that has been difficult.”
**Q25**	Thailand *(#7*, *male*, *63*, *Retired)*	“I normally see my son and the grandchildren once a week. Since COVID-19, they cannot come to see me. But I still talk to them on the phone.”
**Q26**	Malaysia *(#11*, *male*, *52*, *Business manager)*	“My father passed away due to lung cancer during MCO. The hospital did not allow visits by family members, so they did not get to see him in his final days… Friends, relatives and family members could not cross state borders to attend the wake to pay their final respects.”
**Q27**	UK *(#7*, *female*, *48*, *Support worker)*	“I also get support from alcoholics anonymous. But those meetings have also come to an end because of COVID. So, it’s been really difficult. […] Without other sources of support, I’m really struggling.”
***Workplace related challenges and income loss***		
**Q28**	Thailand *(#16*, *f1 female*, *49*, *Masseuse)*	“The [massage parlour] has been closed since March. I completely have not had any income since then, but I have all the same regular expenses–rent, food, children go to school. We are on daily wage. If we don’t work today, we struggle the next day. I am using my saving at the moment.”
**Q29**	Italy *(#10*, *female*, *64*, *Psychologists)*	“Those who suffered the most were those who had precarious situations both economically and psychologically, the most fragile people are those who suffered the most. The government must help those who are really in trouble. Perhaps the only mistake was the lack of timeliness in helping these more fragile people who, because of COVID, lost that job that was already precarious.”
**Q30**	Thailand *(#8*, *male*, *20*, *University student)*	“We have been turning to online learning. I had to drop out from the course as I don’t have high speed internet at home. It is too costly. We cannot afford it. If I could have enrolled in those online courses, I could have graduated this summer.”
**Q31**	Malaysia *(#16*, *female*, *37*, *Nurse)*	“We are working, I think, day and night, so every day we are working extra hours. It is a busy life, always about work and work. Even weekends we also have to come to work.”
**Q32**	UK *(#3*, *female*, *56*, *Doctor)*	“I can’t think how tired I am at the end of a day’s work, its much, much more tiring than our normal way of working. . . In normal times it’s a quick triage to see if somebody needs to be seen to be assessed or whether we can just advise over the phone, but they are turning into full phone consultations, so taking a lot longer … [because I’m] also weighing up the risks of bringing someone into the practice. […] Medico-legally it’s much more risky than our normal [consultations] and there is always that added stress there of missing something."
**Q33**	UK *(#5*, *male*, *33*, *Doctor)*	“People asking how I am, assuming that I would give a horror story from the battlefield. I am not personally involved with coronavirus patients, like the images that people have seen on the news. I was feeling a bit guilty that I’m less stressed than I’ve ever been. . . I have colleagues who have a different experience. Senior colleagues who have been redeployed to do donkey work on maternity or emergency wards. Now they are doing 12-hour shifts–consultants who are not used to that normally. So, my experience is my experience. Some other colleagues have seen their day-to-day routine change for the worse.”
**Q34**	UK *(#14*, *male*, *31*, *Doctor)*	“And obviously demographically I also knew from experience in China that as a young person with no comorbidities I would statistically be a very low risk. . . Obviously that would be different if for example if I had an organ transplant, I was on immunosuppressive medication or if I had diabetes or if I was very, very obese… but obviously I consider myself a very healthy person so [with] that sort of risk assessment you subconsciously think you’ll be fine as well.”
**Q35**	Italy *(#11*, *30*, *female*, *Teacher)*	“Before I used to go out, accompany the children to kindergarten, and then going for a run. Now I couldn’t do it anymore and it is challenging because it was a way to release stress. […] My husband is working from home, so we share the morning management of the children. […] Although during the day he is working, so I have to take care of the children by myself.”
***Poor mental health and wellbeing***		
**Q36**	UK *(#18*, *female*, *40*, *Finance officer)*	“. . . For a long time, I have not been out […] I had to nip to the local [supermarket] and I was absolutely worried sick about going into the shop to go and get some groceries. […] And I got so stressed and so anxious going into shop. That’s never happened before. And honestly, I got out got in the car and… I just completely burst into tears and I was really shaking, and I felt such a Muppet because it was only the small [village shop]. And I just absolutely broke down because it’s stressed me out just going into the shop.”
**Q37**	UK *(#18*, *female*, *40*, *Finance officer)*	“…You are in your house and you can’t escape really, you can’t have something to distract you… any problems that you have, whether its grief or anything else, you can’t really escape them or put things into perspective. […] Whereas if you are not in isolation or lockdown, you can, just by going out for a proper walk and talking to a friend or anybody, just face to face or the whole family just go out for a walk or somewhere different, it helps you take that backwards step. Just being contained in the same four walls makes that difficult to do I think.”
**Q38**	UK *(#5*, *male*, *33*, *Doctor)*	“When there was lack of PPE, I used to get quite angry at the government. […] I felt like I was being failed as a health professional by the government. […] One of my senior colleagues ended up in ITU. I don’t even know if he’s still alive. […] I was really stressed about PPE and my colleague getting ill.”
**Q39**	Malaysia *(#16*, *female*, *37*, *Nurse)*	“I mean if you [have] contact with the positive patient then you yourself [may be] affected by the virus. […] I worry about me bringing back the virus to my husband or to my family members.”
**Q40**	UK *(#3*, *female*, *56*, *Doctor)*	"I think I have the double whammy of basically understanding how the pandemic works and how viruses work. . . but for me knowing what my risk factors were and normally I am constantly catching things from patients. . . and I just had this huge anxiety that it would be passed on to me… and that I would end up, because I am in the wrong age group, I am in the wrong BMI, because I have got the wrong underlying health conditions, that I was potentially at greater risk than everybody else."
**Q41**	Thailand *(#16*, *female*, *49*, *Masseuse)*	“The room is too small to keep distance. My daughter cannot stay in the same room with me. She has to rent another room to reduce risk of getting COVID-19.”
**Q42**	Thailand *(#4*, *male*, *31*, *Hotel bar manager)*	“I cannot go home and have to rent a room away from my parents. I am in a high-risk group, and I do not want to increase their risk.”
**Q43**	Italy *(#11*, *female*, *30*, *Teacher)*	“I was scared when I heard the news about the coronavirus-related child syndrome.”
***Coping strategies***		
***Financial coping***		
**Q44**	Thailand *(#14*, *female*, *45*, *Bus driver)*	“As a tour bus driver, I haven’t had any tour group come in over the past two months. I have to find whatever way to earn money. I have been working for [a food deliver service]. It is not much but at least it is enough for my personal expenses. I have to plan for the next two years which I am not sure whether the situation will improve.”
**Q45**	Thailand *(#9*, *male*, *49*, *Taxi driver)*	“I normally drive my taxi at night. Since the government announced the curfew and people cannot be outside after 11pm, I have changed to rent a taxi to drive from morning until midday, have a break as it is too hot and no passengers are outside, then I go out again in the late afternoon and finish around 8.30pm, then return the car and go home.”
**Q46**	UK *(#9*, *female*, *59 years*, *Unemployed)*	“For some people on benefits, they were given an extra £20 a week. But the benefits we’re on, we didn’t get any more money [. . .]. Money is always a worry for us. When you’re not working, and living on benefits, money is always a struggle.”
**Q47**	Thailand *(#3*, *male*, *26*, *Professional boxer)*	“[During the lockdown], I decided to go back to my hometown to do farm work. We have eggs, rice, fish, and vegetables. We don’t need to always use money like when living in Bangkok.”
***Psycho-emotional coping***		
**Q48**	Thailand *(#16*, *female*, *49*, *Masseuse)*	“COVID effects everyone. It teaches us from to be strong from weakness, to be brave from fear. […] Families, friends and our loved ones are my support. I also meditate and pray. These things make me feel more relaxed and clearer minded. If I stay home and just eat and do nothing, it is stressful. With meditation and prayer, I feel two months of COVID have passed so quickly.”
**Q49**	Italy *(#9*, *49*, *male*, *Handicraft worker)*	“Managing the day at home was interesting, if you wanted there was space to do other things. I painted and worked in the garden and the time passed quickly.”
**Q50**	Malaysia *(#3*, *male*, *52*, *Customs officer)*	“I lost my social life, but I’m gaining time with my family, catching up with some movies, good, good movies. […] Some of my friends have picked up cooking from YouTube. They learn cooking from YouTube.”
**Q51**	Thailand *(#18*, *female*, *45*, *Dentist)*	“It’s like “Plong” [acceptance]—we have a better understanding of the Buddhist Dharma [cosmic law and order], that everything is impermanent–arising, staying and disappearing. Life is impermanence. We cannot change those outside circumstances.”
***Social coping and connectedness***		
**Q52**	Malaysia *(#16*, *female*, *37*, *Nurse)*	“I think the only thing that keeps you sane is your phone […] texting and calling family members and all. [Staying] in touch.”
**Q53**	Italy *(#2*, *female*, *26*, *University student)*	“I called friends on video call, I discovered a way to see those far away, we met more frequently.”
**Q54**	Italy *(#1*, *female*, *50*, *Researcher)*	“I wanted to do something for society, I thought about it and I found a way to be useful and this was a relief. From a moral perspective I strongly felt this urgency. I offered ethical support to my physicians’ friends, for their ethical dilemmas.”
***Reducing and mitigating risks***		
**Q55**	Malaysia *(#16*, *female*, *37*, *Nurse)*	“I worry… but then as long as you practice the right things… [that will] lessen the worry. […] …as long as you take good measure about how to protect yourself, in such ways, your action will save you from getting it.”
**Q56**	Thailand *(#5*, *female*, *41*, *Nurse)*	“I asked my colleague to spray alcohol over my PPE form, wait until the alcohol is evaporated and then I take it off. When I get home, I wash myself thoroughly before I go to my mum’s house to pick up my child.”
***Limiting exposure to the news***		
**Q57**	UK, *(#10*, *female*, *56*, *Photographer)*	“I’m pretty sensitive so how we managed it was too to shut off. […] We shut everything off and I just got the information we needed to keep us safe. […] being able to shut down from all media and everything was really, really helpful because I just wanted to make sure that we weren’t living in fear and sorrow and terror and confusion.”
**Q58**	Thailand *(#10*, *female*, *73*, *Retired)*	“Right now, I have stopped watching news. Following the news gives me too much stress, l watch people fighting to stock their food supply, and people in queues to receive free items. These pictures make me feel sad.”

### Views on COVID-19 public health measures

Across the four countries, participants held complex and nuanced views about government imposed COVID-19 NPIs, which varied depending on participant’s personal circumstances, concerns and goals (e.g., staying safe/at home vs maintaining income/continuing to go out to work), and the extent to which NPIs were seen to facilitate or hinder these goals.

#### Agreement with government response

Across countries, those who largely agreed with NPIs said that the measures, albeit being strict, were necessary to prevent community spread of COVID-19 (see Q1, Thailand). Many expressed feeling safer as a result of imposed NPIs. Especially those with underlying health conditions who considered themselves at high-risk of COVID-19, or who lived with vulnerable family members were generally supportive of strict government measures to curb the spread of COVID-19. Some said their government had handled the pandemic well compared to other countries, thus deeming NPIs to be effective (see Q2, Malaysia). Such views were sometimes linked to narratives about the government doing their best amidst challenging circumstances and emphasised a sense of unity among the people, who found themselves “all in the same boat” (see Q3, Italy).

#### Disagreement with government response

Disagreement with government handling of COVID-19 ranged from mild criticisms about certain aspects of NPIs to some participants saying their government had failed to respond effectively. These participants believed that current NPIs were insufficient and "not strict enough" (see Q4, Malaysia) to contain further spread. For example, some said their government had done too little and “locked down way too late” (see Q5, UK), that rules lacked enforcement by the police and criticised premature easing of restrictions, raising fears about a “second wave” (see Q6, UK). Furthermore, in the UK, some participants who considered themselves at high-risk of COVID-19 infection criticised the government’s ‘shielding list’ for being limited and therefore failing to offer protection to vulnerable populations in line with scientific evidence (see Q7, UK).

Contrarily, others felt that NPIs were too strict “because people have been pushed like animals into cages” (see Q8, Italy). Some Italian participants also believed that measures should have been implemented regionally, not nationally. In Thailand and Malaysia, some participants said that measures like curfews, travel restrictions and boarder closures threatened livelihoods, such as in tourism and for migrant workers, and that financial support from the government was insufficient (see Q9, Thailand; Q10, Malaysia). Particularly those working in the informal economy, such as taxi drivers, sellers, and entertainment workers, expressed significant concern about the impact of NPIs on their financial security, saying that the measures restricted their ability to work.

In Thailand, some participants also criticised certain government measures for furthering the spread of COVID-19, such as the lockdown of Bangkok, which caused thousands to travel to their rural hometowns and the government financial support scheme, which caused large crowds to gather outside designated banks (Q11, Thailand).

Many participants also reported confusion and a lack of clarity in relation to NPI rules and regulations in their country. Particularly participants from Italy and the UK–and to a lesser extent those from Malaysia and Thailand–commonly voiced frustration about the “mixed messages” and lack of clear NPIs guidelines from their governments, leaving many to feel unsafe and a lack of trust in their government’s ability to respond to the pandemic threat (see Q12, UK, Q13, UK).

### Lived experiences and impact of public health measures

**Fig 2:**
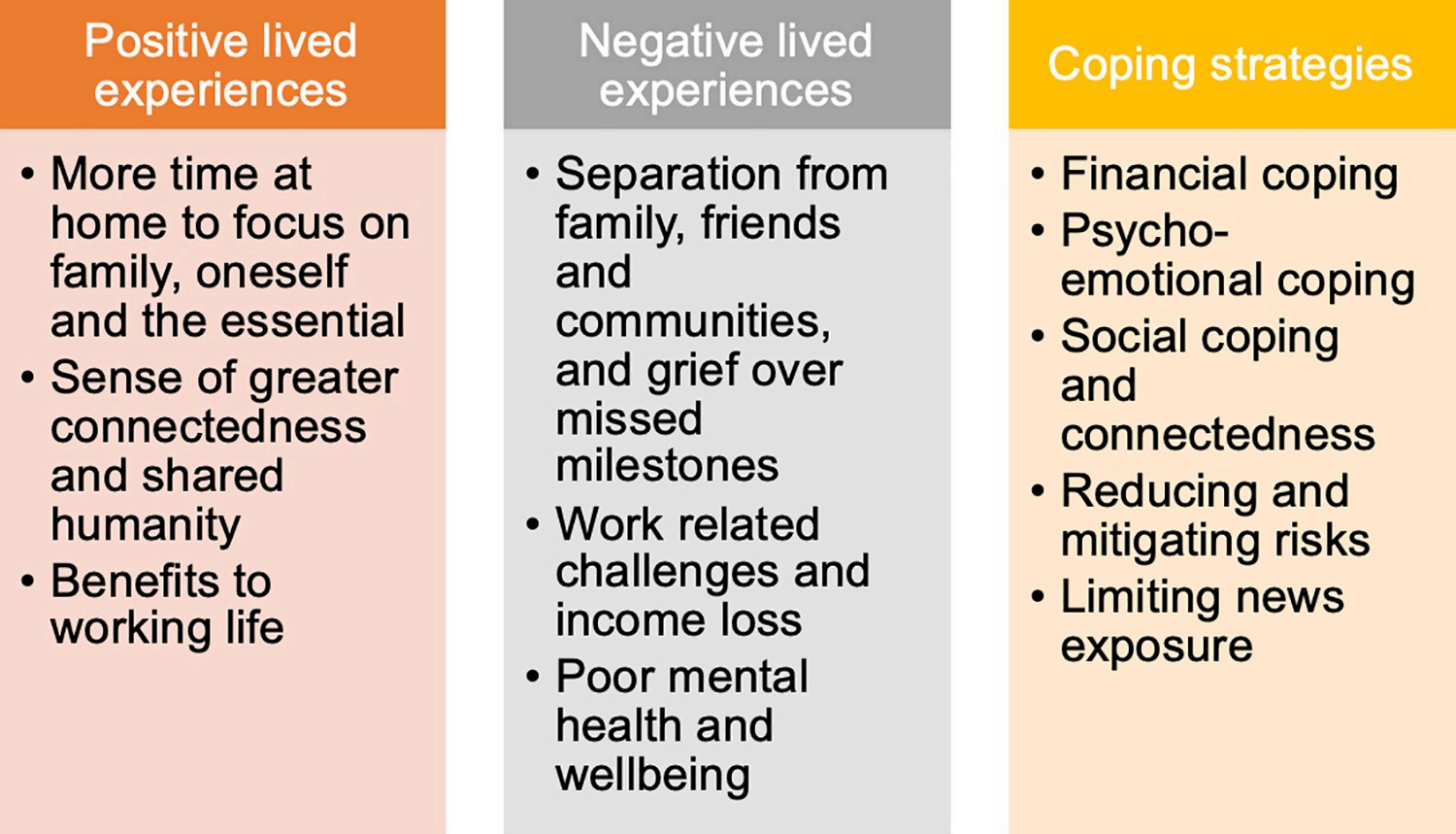
Lived experiences and impact of living under COVID-19 public health measures in four countries. Summary of key positive experiences, negative experiences and coping strategies.

Most participants reported that government imposed NPIs impacted their lives in negatives ways. However, many also discussed inadvertent positive experiences, opportunities or ‘silver linings’, which had resulted from the COVID-19 measures. The extent of both positive and negative experiences varied considerably between participants, based on personal circumstances, particularly financial security, and across countries. Participants also discussed coping strategies adopted to mitigate the negative impacts of the measures. Fig 2 shows a summary of key findings.

### Positive experiences

#### More time at home to focus on family, oneself and the essential

Having more time to spend at home was a key positive experience reported across the four countries. Many compared the constant busyness of their ‘normal’ lives with the quiet and stillness during lockdown, prompting them to focus on what they felt was most essential in life. For some, this facilitated more time with family, extended parental leave, helping children with schoolwork, or reconnecting remotely with family and friends (see Q14, Italy). Others appreciated having more time to themselves, learn new skills, rest and reflect, engage in spiritual practice, or establish a healthier lifestyle, through exercise and cooking. Some enjoyed feeling less pressure to socialise and also said they “save[d] a lot of money” while staying at home (see Q15, Thailand).

#### Sense of greater connectedness and shared humanity

Across countries, some participants reported experiencing a greater sense of social support and solidarity within their communities during lockdown. Many said local support and volunteering initiatives that had sprung up in light of the community coming together, saying that the crisis had triggered a greater recognition of being “interconnected*”* and a sense of shared humanity (see Q16, Italy). Neighbours, religious and community-based organisations were said to be important sources of daily support during local lockdowns, especially for older people and those in need of assistance (see Q17, Malaysia). In Thailand, several participants said that communal food pantries were being set up across the country to help those in need and reflected on how pandemic restrictions had brought out the Thai cultural virtues and values of kindness and generosity (‘*namjai’*) (see Q18, Thailand). Some participants in the UK and Italy additionally noted that the pandemic had made them feel more connected and appreciative of “the beauty of nature” (see Q19, Italy).

#### Benefits to working life

Some participants said that the measures had had a positive impact on their working lives. Across countries, some participants welcomed more flexible hours, home working, the lack of or reduced commute, with less traffic and pollution, while some also reported increased productivity and decreased work-related stress (see Q20, UK; Q21, Thailand). In a few deviant cases, participants also mentioned new business opportunities resulting from restrictions, such as setting up food delivery businesses at home (see Q22, Thailand). Some HCW reported experiencing a greater sense of appreciation and recognition from their patients and society more general, compared to pre-pandemic times (see Q23, UK),

### Negative experiences

#### Separation from family, friends and communities, and grief over missed milestones

Participants across all countries described their separation from friends and family as the most challenging aspect of NPIs. Many felt isolated and lonely due to prolonged physical separation from their loved ones. This was emphasised especially by participants who lived alone, who–unlike those living in larger households–emphasised the importance of human touch and missing physical contact with their loved ones after prolonged periods of isolation (see Q24, UK). Notably, many of those who reported feeling isolated and lonely were older people, who often lived alone (see Q25, Thailand).

Feelings of separation and isolation were described as most challenging when participants were unable to communally celebrate significant milestones, such as birthdays, weddings, religious ceremonies, holidays, or funerals (see Q26, Malaysia). Across countries, many described a feeling of ‘missing out’ on important life events, with this being particularly poignant among older and younger participants, for instance around missing out on experiencing grandchildren’s development or being involved in activism. These finding suggests that the value and grief that participants ascribed to the time ‘lost’ due to NPIs differed across age groups, with the oldest and youngest appearing most negatively impacted by the restrictions.

Furthermore, the closure of recreational facilities, such as cinemas and gyms, or social support services and community organisations, such as mental health support groups or religious services, was perceived as particularly challenging by some (see Q27, UK).

#### Work related challenges and income loss

Many participants across countries described the negative impacts of NPIs on their work. Thai and Malaysian participants, in particular, and to a lesser extent UK and Italian participants, said that the fear of, or actual loss of their job or income was a grave consequence of the restrictive measures (see Q28, Thailand). Particularly those working in the informal economy Thailand and Malaysia–like those working in the service industry as taxi drivers, sellers, and entertainment workers–expressed significant concern and fear about the impact of NPIs on their financial security. For example, some described that travel restrictions and curfews caused pressure on their work schedules and livelihoods (see Q45, Thailand). Income related challenges were especially emphasised by those who lacked savings and were unable to shift to online- or home-working, with these tendering to be participants with lower levels of education and working in lower skilled jobs in the informal sector.

Some participants also worried about the impact of NPIs on society and the economy more broadly, expressing particular concerns about mass unemployment and negative consequences for those in “precarious situations, both economically and psychologically” (see Q29, Italy).

Many who continued working reported challenges due to their altered work situation, including challenges related to poor internet connectivity (see Q30, Thailand), difficulties concentrating while home-working, increased workloads, and heightened work-related stress and anxiety. Particularly HCW reported experiencing greater work related stress due to increased workloads, extended working hours and heightened responsibilities related to the increased pressure posed by COVID-19 on the healthcare system (see Q31, Malaysia; Q32, UK). However, the extent to which HCW reported feeling overwhelmed appeared to be determined by several factors, including workload (see Q33, UK), as well as their level of exposure to affected patients and their own perceived risk-level (see Q34, UK).

Some young professionals expressed concerns about disruptions to their career progression. Finally, women in particular described the increased burdens of unpaid care and domestic work during lockdown (see Q35, Italy). This was more pronounced among participants caring for younger children and children with special needs or living with disabilities (e.g., autism), in the absence of in-person support services or help from relatives.

#### Poor mental health and wellbeing

The negative impact of lockdown measures on mental health and wellbeing was a consistent theme across countries. Several participants reported low mood, grief, fatigue, restlessness, boredom, guilt, feeling overwhelmed and a lack of control as a result of the changes to their personal, family and work lives. For many participants, concerns about contracting COVID-19 resulted in strong fear and anxiety, including around leaving the house or doing shopping (see Q36, UK). At the same time, some described how being stuck at home made it difficult to “escape” these negative thoughts and feelings or “put things into perspective” (see Q37, UK).

Reports of COVID-related stress and anxiety were heightened among those who considered themselves or a family member to be vulnerable, and among HCW, who considered themselves at high-risk of exposure to the virus through their work. Frontline HCW frequently criticised the lack of personal protective equipment (PPE) or changing PPE guidelines, causing considerable fears about their own and their household member’s health (see Q38, UK; Q39, Malaysia), particularly among those with underlying health conditions (see Q40, UK). Participants in Thailand and Malaysia living in large households, but often with limited space, reported heightened fears and worries about spreading COVID-19 to their household members (see Q41, Thailand), particularly when living in multi-generational households with older relatives. This caused some to temporarily relocate to rented accommodation away from vulnerable household members, in turn creating additional financial strain on the family (see Q42, Thailand).

Future uncertainties resulting from NPIs, such as when and how the measures would be eased and the impact on job prospects, was reported as a major stressor on mental health. Parents also commonly worried about the long-term impact of the measures on their children (see Q43, Italy).

### Coping strategies

Across countries, participants reported various ways of coping with the impacts of government imposed COVID-19 NPIs.

#### Financial coping

Many participants described their efforts to cope with financial challenges and income loss resulting from COVID-19 restrictions. For example, some participants tried to reduce spending where possible, while others tried to increase income, such as by starting a home business or taking on additional jobs (see Q44, Thailand).

In Thailand and Malaysia, informal sector workers described adapting working schedules around imposed curfews and travel restrictions (see Q45, Thailand). Participants in the UK, Italy and Thailand, who received financial support from their governments described this as helpful but insufficient to make up for lost income (see Q46, UK, Q9, Thailand). Some Thai participants who normally worked in urban centres reported moving back to their home province where they could live more cheaply, as a way of reducing their financial insecurity resulting from NPIs (see Q47, Thailand).

#### Psycho-emotional coping

In adapting to the ‘new normal’, many participants actively tried to maintain wellbeing amidst difficult life circumstances, with similar coping strategies being reported across countries. These included engaging in religious and spiritual practices (e.g., meditation, prayer) (see Q48 Thailand); establishing daily routines around self-care, work and childcare (e.g., regular exercise, sleep, getting dressed as if going to work, home schooling schedules); maintaining relationships, social connections and recreational activities from home (e.g., using online media) (see Q49, Malaysia); and making home improvements (e.g., tidying, doing home repairs, gardening) (see Q50, Italy).

As a way of coping, some Thai participants, in line with Buddhist principles spoke about intentionally trying to cultivate attitudes of ‘acceptance’ and ‘letting be’ (i.e., “*Plong*”), rather than focusing on the uncertainty surrounding pandemic restrictions (see Q51, Thailand).

#### Social coping and connectedness

Across countries, staying connected with friends, family and peers through shifting social routines online and via telephone was considered essential for keeping “sane” during prolonged physical separation from loved ones and for maintaining a sense of connectedness (see Q52, Malaysia; Q53 Italy).

Some participants felt that a renewed sense of solidarity and communities coming together helped to counter feelings of loneliness and isolation. Several reported “want[ing] to do something for society” (see Q54, Italy), saying that volunteering initiatives or providing personal support to family, friends, neighbours and colleagues helped them to experience a sense of purpose during this difficult time.

#### Reducing and mitigating risks

Across countries, many participants reported taking steps to reduce their risks of potential exposure to COVID-19, including by staying at home; switching to online grocery shopping or designating one person per household to do in-person shopping; using PPE like hand sanitiser, gloves, face masks and shields; and avoiding close contact with others when in public. Particularly HCW reported that having access to PPE reduced fears and increased their sense of control (see Q55, Malaysia; Q56, Thailand).

#### Limiting exposure to the news

Across countries, many participants reported feeling distressed by the constant exposure to COVID-19 news, which some felt were unclear, untrustworthy and politicised. As such, in trying to balance their desire to stay informed and to manage the anxiety evoked by the news, many participants said they deliberately limited their daily news exposure (see Q57, UK; Q58, Thailand).

## Discussion

This study provides insight into the lived experiences of government imposed NPIs among the public, including HCW across four countries during the first wave of the COVID-19 pandemic, an unprecedented time in history. Despite considerable variation of individual circumstances, there were some surprising similarities between participants’ experiences across countries, including around personal, social, familial, professional, financial, religious and spiritual life domains. This study unusually highlights the positive silver linings and coping strategies that individuals adopted, alongside the better understood negative psychosocial and economic impacts of COVID-19 related NPIs.

Across countries, participants generally had strong views, either supportive or critical of their government’s handling of the pandemic, with few expressing neutral views. Those agreeing with their government’s response to COVID-19 emphasised the necessity of NPIs to prevent COVID-19 transmission, appealing to values of unity and solidarity. Criticisms of the government’s response emphasised that NPIs were either not strict enough (e.g., leading to fears about COVID-19 transmission) or too strict (e.g., threatening livelihoods and income security). Overall, many expressed confusion and frustration at rapidly changing rules, echoing other research linking negative views of NPIs to mistrust of politics and negative perceptions of government transparency [20, 64].

Notably, participants from Italy and the UK were most outspokenly critical of their government’s handling of the pandemic than those in Malaysia and Thailand. A cross-country study conducted among 19 countries during the COVID-19 pandemic showed geographic differences in the trust that individuals had in their government’s ability to successfully address unexpected health threats [65]. Other research has shown that levels of trust in governments in East Asian countries are generally higher due to their authoritarian orientations and leadership, which function as a historical-cultural source of political trust [66]. Trust in government has been associated with higher levels of public compliance with public health measures during the COVID-19 pandemic [67], and during past outbreaks of Ebola [68] and H1N1 [69, 70], which may help explain why COVID-19 transmission was initially more contained in Malaysia and Thailand and why participants from Thailand and Malaysia expressed less disagreement with their government’s pandemic response.

However, in interpreting these findings, several other factors need to be kept in mind. Firstly, at the time of interviewing, Italy and the UK had higher COVID-19 case and mortality rates compared to Malaysia and Thailand [1], with comparative research showing that population risk of exposure (i.e. country level COVID-19 mortality rates) correlate with public perception of government responses to COVID-19 [65]. Furthermore, differences in the state of democracy across the four countries [71] may also have influenced respondents freedom to express disapproval and dissenting opinions about their governments during the research interview.

With regards to the lived experience and impacts of public health measures, most participants felt predominantly negatively affected by NPIs, including through income loss, work related challenges, separation from loved ones, grief over missed milestones, and widespread experiences of poor mental health. This supports existing qualitative evidence from the UK highlighting the negative impacts of social distancing and isolation, including economic, social, psychological, and emotional losses, such as loss of motivation, meaning and self-worth [20]. While the adverse impacts of NPIs on mental health were emphasised by participants across countries, participants from Malaysia and Thailand reported particularly high concern and distress with regards to the economic impacts of NPIs. This finding could be explained by the higher proportions of informal sector workers in low- and middle-income countries (LMICs), who are less able to shift to telework, have fewer savings and lack social protection, and are therefore less able to absorb the economic shocks of NPIs like travel restrictions and closure of non-essential businesses [12]. These findings highlight marked cross-country differences in the capacity of individuals to bear the burdens of prolonged lockdowns and reduced economic activity, underscoring the importance of government financial support schemes for those economically most disadvantaged by NPIs. While governmental financial support strategies were in place in all four countries included at the time of this study, most participants who received government support felt that this was insufficient. This suggests that while countries have implemented a range of strategies to reduce the financial hardships resulting from NPIs, further support to help low-income households is still needed.

Our analysis of lived experiences of COVID-19 NPIs also suggests that socio-political and health systems contexts intersect with factors including economic precarity, living arrangements, age, gender, and COVID-19 risks, to shape individuals’ vulnerability to the negative socio-economic and psychological impacts of NPIs. For example, participants in this study who lived in larger households often expressed greater fears about contracting or transmitting COVID-19, which echoes research showing that those from poorer families and ethnic minorities are more likely to live together in crowded housing, thereby increasing their risk of COVID-19 infection [72, 73]. Differences in COVID-19 related fear and anxiety could further be explained by the different contexts of health systems, which largely underpin the availability, accessibility, affordability, and quality of healthcare services in each country.

Additionally, while it is well known that older people are physically more vulnerable to COVID-19 [74], older participants in our study more often reported feeling lonely and isolated, suggesting that older people may be particularly vulnerable to experiencing psychological distress in response to NPIs. Furthermore, lower rates of literacy and technological skills among the older population are likely to create additional barriers for maintaining social contact, and accessing healthcare and social support services remotely when NPIs like movement restrictions are in place [75, 76]. Challenges facing older people during COVID-19 lockdowns are likely to be exacerbated for those living in LMICs, like Malaysia [75] and Thailand [77], where older people commonly live in multi-generational households (thereby increasing their risks of COVID-19 exposure), and where greater health system level barriers exist for accessing age-appropriate services and support [76, 78]. Governments should attend to the specific challenges facing older populations, such as by adopting strategies to tackle isolation and loneliness, such as the UK’s “support bubble” intervention introduced in September 2020 [79] to support health and wellbeing of older people who live alone in light of ongoing pandemic restrictions.

Moreover, gender represents another important social dimension underpinning the differential lived experience and impact of NPIs within and across countries. In this study, several women described struggling with the added burden of care work due to prolonged closures of day-care centres and schools during lockdown. This is unsurprising given that women provide 80% of unpair labour globally, much of this care work [80]. Women in LMICs face additional challenges, owed to the fact that two thirds of informal sector workers are female, thereby making women more likely to lose their income through COVID-19 NPIs [81]. Together, these trends suggests that women, particularly those in LMICs, are disproportionally affected by NPIs, and that this is likely to have far reaching consequences on their long-term mental health and economic security. Policy makers therefore need to ensure that NPIs do not further exacerbate gender inequalities.

Importantly, this study has shown that in the context of NPIs, socio-economic factors like poverty, occupation, gender, age, and healthcare access intersect to further entrench existing inequalities in society. This suggests that the risks and burdens of NPIs, and the access to resources to mitigate their impacts and prevent the spread of COVID-19 are distributed unequally within and across populations. Our findings thus emphasise that in considering ways forward in this ongoing pandemic, governments need to carefully consider those multiply disadvantaged and vulnerable to experiencing the adverse consequences of NPIs.

Furthermore, with regards to HCW, research from other epidemic contexts, like SARS, Ebola and MERS have highlighted that–in additional to physical health risks–HCW are particularly prone to psychological harms, like trauma or stress-related disorders, depression, and anxiety, as a result of health emergencies [82]. Our findings support others in showing that negative experiences were often amplified among HCW who faced greater work-related stress and exposure to COVID-19 risks [22, 23, 25, 83]. This study also highlights that experiences among HCW differed depending on their own levels of self-perceived COVID-19 risk, which appeared to be largely shaped by their own health status and the context of their daily work. This resonates with research documenting significantly higher levels of COVID-19 infection among patient-facing HCW, compared to non-patient facing HCW [84]. Other research that has investigated the psychological impacts of COVID-19 on HCWs from various socio-demographic backgrounds (e.g., age, gender, place of work) and healthcare specialities (e.g., doctors, nurses and other HCWs), showed that particularly younger, female HCW, nurses, and those working in higher-risk areas and with poor social support were the most likely to face mental health problems [82, 85, 86]. This suggests that for HCW employed in contexts which experienced funding cuts and workforce shortages prior to the pandemic, such as the UK [87] and those in LMICs, where shortages of HCWs and poorer healthcare infrastructure is common, these impacts are likely to be worse. Together, the trends suggest that–like the public–HCWs also face differential vulnerabilities to the negative impacts of COVID-19 and related NPIs, depending on the convergence of salient contextual and individual-level factors. These differences–both between and within countries–need to be considered when developing tailored interventions to support the global healthcare workforce in the face of this pandemic [88, 89]. In order to be effective, such interventions need to address both individual level coping and resilience (e.g., promoting self-care and positive mental health) as well as structural and institutional contexts (e.g., instrumental support like PPE; informational and organisational support, like training, guidelines and managing workloads; and mental health support) [90].

Finally, our findings have highlighted inadvertent positive experiences and coping strategies emerging from this crisis, which have been largely overlooked by research to date. Key positive experiences discussed in our study included participants having more time to focus on their family, themselves and what they most valued in life; making positive changes to working routines; and feeling a greater sense of community. Many participants across countries rapidly adapted their lives by developing financial, psycho-emotional and social ways of adjusting to the ‘new normal’, and by reducing COVID-19-realted risks and exposure to anxiety provoking news. However, socio-cultural, economic and other contextual factors importantly underpinned the extent to which participants were able to employ positive coping strategies. During this pandemic, some groups, including younger people, sexual and gender minorities and those with financial instability have been evidenced to use less productive coping strategies, like substance use and behaviour disengagement [91]. We support others who argue that, in order to ensure that COVID-19 interventions are equitable, the definition of who is considered vulnerable and ‘most-at-risk’ should be expanded to include social and cultural—not just biological—factors [92–94].

Across countries, community support and solidarity were highlighted as helpful for coping with the psychosocial impacts of prolonged physical distancing and isolation. Public health messaging that emphasises shared values of solidarity, altruism and social responsibility is thus likely to enhance public acceptability of and commitment to NPIs, as has been shown in the case of promoting the uptake of face coverings across several countries [95]. Our findings show that coping strategies employed by participants included both individual level, as well as community and societal level strategies, with some coping strategies reflecting specific sociocultural and religious contexts (e.g. Thai participants drawing on Buddhist principles of ‘acceptance’). Further research is needed to better understand the lived experiences of coping with NPIs and resilience strategies across different socio-cultural contexts in order to develop interventions that can promote resilience at the individual level and strengthen protective factors at community levels, including social networks, to help mitigate the adverse impacts of NPIs [96].

Importantly, understanding positive experiences and coping—and thereby shedding light on the full spectrum of lived experience—can illuminate and facilitate the public’s resilience in light of ongoing restrictions. Findings from our study suggest that efforts to maintain mental health services, community and religious support groups remotely; ensuring availability of PPE; and offering financial compensation for lost income are critical ingredients to support better coping across countries. Furthermore, identifying socially vulnerable groups in each context and accounting for particular socio-cultural needs, through community engagement and social science research, will be essential for policy makers to promote positive coping strategies and reduce the negative and unequal impacts of NPIs [19].

### Limitations

This study is limited in three main ways. Firstly, although we aimed to sample a diverse group, there was less variety in socio-economic status than desired. This was due to rapid recruitment, and interviews being conducted online or by phone, restricting access of those from lower socio-economic groups with less access to these technologies. Additionally, due to data protection regulations, only Microsoft Teams was approved for online data collection in this study, ruling out more accessible and widespread platforms like WhatsApp or Facebook Messenger, likely to have facilitated greater diversity among participants. Despite this, through purposive sampling we managed to include participants from a range of age groups, occupations, educational backgrounds and self-perceived risk levels, including HCW. Secondly, social desirability bias [97] may have led participants to represent themselves as overly adherent to and supportive of NPIs, and avoided revealing more extreme views (e.g., beliefs in conspiracy theories). Thirdly, while this study compared lived experiences of NPIs across countries, future research would benefit from investigating intra-country differences, including comparisons between urban and rural residents [98].

A strength of this study is that it is the first cross-country qualitative study to provide evidence on the impact of NPIs on lived experience of the public, including HCW, which can help to inform the COVID-19 policy response. Additionally, the use of remote interviews allowed for nationwide recruitment across the four countries, circumventing barriers of geographical distance, while also potentially offering a greater sense of safety and anonymity, which may have increased participation from individuals who may be reluctant to participate in-person.

## Conclusion

Findings from this study underscore the need for COVID-19 policies and interventions to pay greater attention to the differential impact of government imposed NPIs on different population groups, as some populations are disproportionally affected by the adverse impacts of NPIs. Supporting individuals’ coping is an important strategy for mitigating the adverse impact of NPIs and interventions should focus on socially vulnerable groups who experience greater negative impacts and are less able to cope. These are likely to include individuals facing multiple socio-economic disadvantages and other marginalised populations, such as sexual and gender minorities and migrant workers. We emphasise that across countries, declines in mental health as a result of NPIs appear widespread and common. Efforts to counter this trend are thus critical: policy makers must prioritise populations physical health—i.e., protection from COVID-19 infection—while not losing sight of their mental health.

## Supporting information

S1 File Interview guide. (DOCX)
