# Supplementary material for: The impact of COVID-19 non-pharmaceutical interventions on the lived experiences of people living in Thailand, Malaysia, Italy and the United Kingdom: A cross-country qualitative study
Source: PLoS One. 2022 Jan 21;17(1):e0262421. doi: 10.1371/journal.pone.0262421 (PMC8782407; doi:10.1371/journal.pone.0262421)
Supplement: S1 File — (DOCX) [file pone.0262421.s001.docx]

**SEB-Cov Qualitative study: Standardised topic guide for Thailand, Malaysia, Italy and UK**

**Review study information and answer any outstanding questions**

**Opening questions**

1. Can you tell me about yourself?
   1. *Probe: age, job, family/living situation*
2. How are you feeling today?
3. (Can you tell me how life in the last weeks has been for you?)

**Response to social isolation/ social distancing/ travel restrictions/ quarantine**

1. What do you think about the measures that your government has put in place to deal with coronavirus?
   1. *Prompt (to support reflection, if needed): These measures include social-isolation, social distancing, travel restrictions and quarantine.*
2. Among all the measures that you mentioned, which measure has affected you the most^[[1]](#footnote-1)^?
3. How have the government’s measures affected you and your life?
   1. *Prompt (if needed): Can you give me an example of any positive effects that you have experienced? (e.g. more time with family/children, less time commuting, doing more regular sport/walks outside etc.);*
   2. *Prompt (if needed): Can you give me an example of any negative effects that you have experienced? (e.g. lost wages, challenges with childcare, food and household supplies, loneliness etc.)*
4. Have you made changes to your daily life to cope with the new measures?
5. Have you faced any difficult decisions over the last weeks?
6. Is there something that would help you cope better with the current situation?
   1. *Probe: Anything else that could help you cope?*
7. How do you think your friends and family are responding to the new measures?
8. How do you think other people in the general public are responding to the new measures?
9. Were there times when you weren’t able or didn’t want to self-isolate/practice social distancing/adhere to travel restrictions/quarantine^1^?
10. If you weren’t able to self-isolate/respond to the other measures^1^, what did you do?

**Wellbeing and mental health**

1. How have you been feeling over the last weeks?
   1. *Prompt: Do you feel fearful about anything in relation to coronavirus?*
2. What has helped your wellbeing and mental health in the last weeks?
3. Is there something you think could help support your wellbeing and mental health in the current situation?

**Information, misinformation and rumours**

1. Where do you get information on Coronavirus?
2. What do you think about the information you receive?
3. How much do you trust different sources of information?
4. Do you have suggestions of how this information could be improved?

**Ending**

1. If there is one thing that you would like to know about Coronavirus, what would it be?
2. If you could have one thing to improve your situation, what would it be?
3. Do you have any other comments that you would like to add?

1. *Note for interviewers*: Only discuss those measures in detail, which the participant has said are relevant to their life (e.g. while social isolation/distancing is likely to affect most participants, travel restrictions and quarantine may not, in which case focus discussions on the former). [↑](#footnote-ref-1)
